# Severe hypoglycaemia in diabetic patients in Pre-hospital and Emergency Department care: a cross-sectional survey

**DOI:** 10.1186/s13104-018-3363-0

**Published:** 2018-04-23

**Authors:** César Esteves, Celestino Neves, João Jaime Sá, Davide Carvalho

**Affiliations:** 1 Endocrinology, Diabetes and Metabolism Department, Centro Hospitalar São João, Alameda Prof. Hernani Monteiro, 4200-319 Porto, Portugal; 2 Faculty of Medicine, University of Porto, Alameda Prof. Hernani Monteiro, 4200-319 Porto, Portugal; 3 Institute for Research and Innovation in Health, Porto, Portugal; 4 Emergency Department, Centro Hospitalar São João, Porto, Portugal

**Keywords:** Hypoglycaemia, Emergency care, Costs, Diabetes mellitus

## Abstract

**Objective:**

We aimed to characterize hypoglycaemia episodes and patients examined by a Pre-hospital Medical Emergency Unit (PH) and in the Emergency Department (ED) of our hospital.

**Results:**

We identified 86 episodes of severe hypoglycaemia (PH: *n* 37; ED: *n* 49; both: *n* 12). Hypoglycaemia accounted for 4.7% of all emergency calls attended by the PH (*n* 793) and 0.11% of all ED episodes (*n* 54,366). Among episodes examined by the PH, 64.5% of involved patients had type 2 diabetes and 54.1% were not referred to the ED. Transportation of the patient to the ED was more likely in type 2 diabetes (p = 0.014). Among episodes evaluated in the ED 66.1% of the patients were more than 65 years old and 81.4% had type 2 diabetes. 66% of the patients were insulin treated. One-third of examined patients were admitted to the ward, the majority having type 2 diabetes.

## Introduction

Hypoglycaemia is a major barrier to the achievement of optimal glycaemic control in diabetes (DM) [1, 2]. It is one of the most common adverse effects associated with antidiabetic drugs and is usually recognized as being potentially harmful to people with diabetes [3, 4]. The ACCORD [5], ADVANCE [6] and VADT [7] trials demonstrated an increase in mortality in the case of intensively-treated patients with type 2 diabetes (T2DM). In analysing the reason for this, one of the main hypothesis is that mortality was associated with hypoglycaemia risk.

ADA [2] reports on the subject consider that severe hypoglycaemia is a condition which requires another person to actively administer carbohydrates, glucagon, or take other corrective actions. In type 1 diabetes (T1DM), the incidence of severe hypoglycaemia is 115 [8]–320 [9] episodes/patient-year, and severe hypoglycaemia may be the cause of death of 4–10% [10] of patients. For individuals with T2DM, the risk of severe hypoglycaemia is lower. It mainly occurs in individuals that have been treated with secretagogues or insulin, in older patients with multiple comorbidities, recent hospitalization and polymedicated patients [2]. Hypoglycaemia in DM has been recognized as a driver for increased costs to healthcare systems.

In Portugal, data regarding hypoglycaemia is scarce [11] as there are no structured databases regarding emergency episodes, and diabetes databases are based on appointment records which do not include information about hypoglycaemia [12]. However, in 2015, an estimated 13.3% [12] of the population had DM and may be at risk of treatment induced hypoglycaemia. The authors intend to evaluate the prevalence of hypoglycaemia and treatment protocols implemented in the Emergency Department (ED) of a leading hospital, and also in the associated Pre-hospital Medical Emergency Unit (PH), as well as the criteria for admission to the ward. We will also look into the causes of evaluated hypoglycaemia episodes.

## Main text

### Methods

We performed a retrospective cross-sectional study of individuals with DM examined in the Pre-hospital and/or ED setting due to hypoglycaemia. Our data refers to episodes that took place between the 1st of January and the 31st of March, 2010, evaluated by the PH or in the ED of our hospital, which provides care in a large city in Portugal. We weren’t able to extend the study period due to software limitations. In Portugal, Pre-hospital Care is usually made by paramedic personnel. Some teams include medical doctors that can treat emergent situations in an outpatient basis, with no need to transport the patient to the hospital. All emergency episodes are documented in the Pre-hospital Emergency Unit file and/or in the electronic health record software used in the Emergency Department–Alert©. We searched the files of the PH and the Alert© software for data on ED episodes, using the ICD-9 codes that are potentially associated with DM and its complications, or hypoglycaemia: 250 (*diabetes mellitus*), 251 (*other disorders of pancreatic internal secretion*), 271 (*disorders of carbohydrate transport and metabolism*), 775.6 (*hypoglycaemia*) and 962 (*poisoning by hormones and synthetic substitutes*). We excluded patients without diabetes, as well as codification errors. We recorded the following variables: demographic data, type of diabetes, antidiabetic drugs, diabetes duration, chronic diabetes complications, comorbidities, presence of neurogenic or neuroglycopenic symptoms, Glasgow Coma Scale and glycaemia changes during the episode. We identified the episodes that resulted in admission to the Short Stay Unit (SSU), admission to the ward and the comorbidities that could be related with the decision to keep the patient in observation. The SSU is a ward where the patient can be kept for observation for less than 24 h, after which it is considered to be an admission to the ward.

We used Microsoft Office 2010 Excel and SPSS 20.0 for statistical analysis. When applicable, we used the χ^2^ and Mann–Whitney tests. The results were expressed as mean ± standard deviation or median [quartiles]. We considered *p *< 0.05 as significant.

### Results

We reviewed 793 emergency calls evaluated by the PH, of which 37 were hypoglycaemia episodes (4.7%). We reviewed 54,366 ED episodes during the study period of which 15,517 were not associated with a definitive diagnosis and therefore were excluded from the analysis. Among the remaining 38,849 episodes, we found 102 episodes, of which 32 were not associated with hypoglycaemia, 8 episodes occurred in patients without diabetes and 1 episode took place in the ED. Forty-two episodes were diagnosed as “other specified hypoglycaemia” and 19 as “hypoglycaemia, unspecified”—totalizing 61 episodes of hypoglycaemia (0.11% of the total ED episodes; 0.16% among episodes with a definitive diagnosis). In total, we identified 86 episodes of severe hypoglycaemia: 37 examined by the PH, of which 12 were referred to the ED and 49 episodes were examined in the ED that had not been previously examined by the PH. The episodes occurred with 84 patients, as 2 individuals had recurrent hypoglycaemia.

#### Pre-hospital Medical Emergency Unit

Twenty episodes (54.1%) did not result in referral to the ED and 13 (35.1%) occurred between 24.00 and 08.00. The characterization of the examined patients is presented in Table 1. People with T1DM were significantly younger than patients with T2DM [40 years (35.0–42.5) vs 75 years (68.5–77.8); *p *= 0.000; Mann–Whitney test], however there were no significant differences in diabetes duration (*p *= 0.203). All individuals with T2DM treated with oral agents only were using sulphonylureas.

**Table 1:** Characterization of patients with hypoglycaemia examined by the PH (*n* 37) and in the ED (*n* 59)

	Pre-hospital Emergency Unit	Emergency Department
Age (years)	Mean ± SD: 60.7 ± 18.36 Min–max: 27–84 > 65 years: 19 (51.3%)	Mean ± SD: 68.0 ± 15.26 Min–max: 23–93 > 65 years: 39 (66.1%)
Gender	Men: 10 (27.0%) Women: 26 (72.3%) *Missing*: 1	Men: 28 (47.5%) Women: 31 (52.5%)
Type of diabetes mellitus	DM1: 11 (35.5%) DM2: 20 (64.5%) *Missing*: 6	DM1: 5 (8.5%) DM2: 48 (81.4%) Other: 6 (10.2%)
Diabetes duration (years)	19.1 ± 8.37 Min–max: 10–30	16.2 ± 10.25 Min–max: 0–37
History of diabetes complications	–	Yes: 35 (59.3%) No: 24 (40.7%)
History of severe hypoglycaemia	Yes: 8 *Missing*: 29	Yes: 12 (20.3%) No: 47 (79.7%)
Antidiabetic agents	Only OA: 5 (26.3%) Only insulin: 9 (47.4%) OA plus insulin: 5 (26.3%) *Missing*: 18	Only OA: 20 (33.9%) Only insulin: 30 (50.8%) OA plus Insulin: 9 (15.2%)

*OA* oral agents, *DM1* type 1 diabetes mellitus, *DM2* type 2 diabetes mellitus, *DM3* other causes of diabetes mellitus

Mean glycemia at presentation was 32 ± 14.9 mg/dL and only one-third of patients referred neurogenic symptoms. There was a previous attempt to treat hypoglycaemia by a relative in six episodes (16.2%). Thirty-five cases were treated on site by a health care professional using hypertonic glucose. There was no reference to use of glucagon. Episodes involving patients with T2DM were more frequently associated with referral to the ED than those in individuals with T1DM [14 (70.0%) vs 3 (25.0%); *p *= 0.027; χ^2^ test]. Refusal of the patient was the reason why 17.6% of patients were not evaluated in the ED.

#### Emergency Department

Table 1 describes the detailed information of patients with hypoglycemia examined in the ED. There was a high prevalence of significant comorbidities, such as chronic kidney disease (*n* 21, 34.4%), heart disease (*n* 20, 32.8%), cerebrovascular disease (*n* 18, 29.5%), neoplastic disease (*n* 8, 13.5%) and dementia (n 7, 11.9%). Details on the use of oral agents can be found in Table 2. Nineteen patients were using sulphonylureas, of which 8 were using glibenclamide, 5 were using glimepiride and 4 were using gliclazide (*missing* 2).

**Table 2:** Use of oral agents in people with diabetes admitted in the ED for hypoglycemia

Subgroups	Drug class combination	*n*
Oral agents only, without SU	+Metformin and DPPI	1
	+TZD	1
	Missing	1
	Subtotal	3
Oral agents only, including SU	Monotherapy	5
	+Metformin	7
	+DPPI	1
	+DPPI and metformin	3
	+Metformin and AGI	1
	Subtotal	17
Oral agents plus insulin	+Metformin	3
	+Metformin and DPPI	2
	+Metformin, DPPI and SU	1
	+Metformina and AGI	1
	+Metformin, TZD and SU	1
	*Missing*	1
	Subtotal	9
	Total	29

*DPPI* DPP-IV inhibitor, *SU* sulphonylurea, *TZD* thiazolidinedione, *AGI* alpha-glucosidase inhibitor

Details on hypoglycaemia episodes in the ED can be found in Table 3. At the time of arrival to the ED, 30 (49.2%) patients were hypoglycaemic (mean glycemia 43 ± 16.4 mg/dL, 1 below 20 mg/dL). Among these, 16 (26.2%) were diagnosed in the ED. None of the patients admitted in the ED whilst hypoglycaemic was previously evaluated by our PH. In 32 (52.4%) patients, the mean maximal glycaemia during the ED stay was higher than 250 mg/dL. There was a statistically significant difference in Glasgow Coma Scale between patients that were admitted in the ED during nighttime and daytime [8.5 (3.0–15.0) vs 15.0 (13.5–15.0) respectively; *p *= 0.046; Mann–Whitney test].

**Table 3:** Characterization of severe hypoglycaemia episodes recorded in the Emergency Department (*n* 61)

Glycaemia (mg/dL)	On site: 38 ± 13.7 (3 below 20 mg/dL) Minimum–maximum: 20–68 ED arrival: 103 ± 80.5 Minimum–maximum: 20–349 Patients evaluated by our PH: 202 ± 82.6
Glasgow coma scale	All episodes: 14.0 (5.5–15.0) Nocturnal: 8.5 (3.0–15.0) Diurnal: 15.0 (13.5–15.0)
Administration of treatment on site	Relative: 15 (24.6%) Health professional: 26 (42.6%)
Type of treatment on site (available information in 24 cases)	Hypertonic glucose: 13 Per os: 10 Glucagon: 1
Type of treatment in the ED	56 (91.8%) patients received any kind of treatment Per os: 2 Hypertonic glucose: 23 (mean 2.2 ± 0.79 vials) Glucose 5–10% solution: 31
Maximal glycaemia during ED stay (mmol/L)	256 ± 90.9 Minimum–maximum: 85–Hi
Workup requested?	Yes: 52 (85.2%) No: 9 (14.8%)
Admission in the short stay unit	Yes: 18 (29.5%) No: 43 (70.5%)
ED stay duration (h)	17.2 ± 1.27
Discharge destination	Home: 40 (65.6%) Other facility: 2 (3.3%) Ward: 19 (31.1%)

Digestive system associated disease was the most common concurrent cause for hypoglycemia (*n* 17, 27.9%), followed by insulin administration error (*n* 8, 13.1%), acute kidney injury (*n* 7, 11.5%), skipped meal (*n* 7, 11.5%) and urinary tract infection (*n* 6, 9.8%). Other precipitants account for 13 episodes (21.3%) and 11 (18.0%) had no identified cause.

#### Patients’ stay in the hospital

Among 19 patients admitted, 2 stayed in the SSU for more than 24 h and the remaining were admitted to the ward. In 19 patients, 18 had T2DM, of which 9 were using oral agents only—all with sulphonylurea—6 were only using insulin and 3 were treated with combined therapy. Of the patients admitted to the ward, 11 presented reasons for admission other than hypoglycaemia: (1) community acquired pneumoniae (*n* 4), (2) worsening kidney function (*n* 5), (3) myocardial infarction or decompensated heart failure (*n* 3), (4) stroke (*n* 1), (5) lower limb gangrene (*n* 1) and (6) social reasons (*n* 1).

Hospital stay median duration was 9 days (minimum 3–maximum 130), according to the type and severity of comorbidities. 17 (89.4%) patients were discharged home and 2 patients died during hospital stay.

#### Follow up after discharge from the ED/ward

Since the time period of data analysis up to September 2013, 15 (25.4%) patients died. The median time lag between severe hypoglycaemia episode and death was 9 months (minimum 4–maximum 21). Patients that died during follow-up had a mean age of 72.9 ± 11.69 years and severe comorbidities. Among these, vascular disease (n 9) and chronic kidney disease (n 7) were particularly prevalent.

### Discussion

We found a significant prevalence of severe hypoglycaemia among patients evaluated by a PH. A study published in 2006 [13] found that hypoglycaemia is the most common reason for emergency calls related to diabetes or antidiabetic therapy, accounting for 51% of evaluations. We confirmed that the frequency of referral to the ED among patients with T2DM is significantly higher than among individuals with T1DM, as previously described [11].

In previous literature, the prevalence of severe hypoglycaemia in the ED is 0.37–0.4% [14, 15]. We found a lower figure, which might be related to the fact that the previous studies took place several years ago, and that the prescription pattern of antidiabetic drugs differed from that of 2010, as well as recommended glucose targets. In Portugal, most new insulins are fully and most oral agents are partially reimbursed.

In one study [13], 93% of patients was using insulin and only 1 patient did not have documented hypoglycaemia in the pre-hospital context. In our series, 54.4% of patients were using insulin (56.3% among individuals with T2DM) and 16 (26.2%) were diagnosed in the ED. It may be that, in our area, patients and their relatives might not have sufficient therapeutic education for the prevention, identification and adequate management of hypoglycaemia episodes. It is necessary to determine what the difficulties are for pre-hospital diagnosis/evaluation of hypoglycaemia.

Regarding hypoglycaemia treatment modality, IV glucose was the preferred treatment while other authors [13] noted a high prevalence of glucagon-use. The same authors found that most patients arrived at the ED still suffering from hypoglycaemia. In our study, nearly half of the patients were admitted in that condition. This may be related to: (1) inadequate pre-hospital diagnosis; (2) referral of patients to the ED by relatives due to symptoms associated with undiagnosed hypoglycaemia; (3) the patient was unable to ingest carbohydrate and did not have access to IV glucose or glucagon. Every patient evaluated by our PH was adequately treated before arriving at the ED.

Regarding the use of hypoglycaemic drugs, insulin seems to be the most frequent agent associated with severe hypoglycaemia. Nineteen patients were using sulphonylureas and, amongst them, 17 were not using insulin.

The frequency of admission to the ward for prolonged observation was 31.1%, whilst Brackenridge [13] found an admission rate of 11%. There are probably regional differences in criteria for admission to the ward. There is often an association with intercurrent illness which may also be a factor responsible for the elevated reported costs of hypoglycaemia.

Donnelly [8] also found a high mortality after severe hypoglycaemia, which is suggestive of its value as a prognostic factor. Despite not being able to determine the cause of death, the high prevalence of severe comorbidities in patients suffering severe hypoglycaemia is of relevance [16].

### Conclusions

We found that many patients aren’t adequately evaluated and treated before being referred to the Emergency Department. Hypoglycemia is a frequent cause for emergency calls and only a minority those is evaluated in the Emergency Department. Most individuals evaluated in this context use insulin.

### Limitations

We found a significant number of ED episodes with unspecified diagnosis, which may arbor a number of incorrectly codified hypoglycaemia episodes. Episodes associated with specified consequences of hypoglycaemia may have been codified without reference to hypoglycaemia. We were not able to determine the prevalence of use of specific insulin treatment regimens. Causes of hypoglycaemia were determined by the treating physician. These results may not be generalizable to patients from other regions.

## Abbreviations

T1DM: type 1 diabetes mellitus
T2DM: type 1 diabetes mellitus
PH: Pre-hospital Emergency Unit
ED: Emergency Department
SSU: Short Stay Unit
